# US Physicians’ and Nurses’ Motivations, Barriers, and Recommendations for Correcting Health Misinformation on Social Media: Qualitative Interview Study

**DOI:** 10.2196/27715

**Published:** 2021-09-01

**Authors:** John Robert Bautista, Yan Zhang, Jacek Gwizdka

**Affiliations:** 1 School of Information The University of Texas at Austin Austin, TX United States; 2 Center for Health Communication Moody College of Communication and Dell Medical School The University of Texas at Austin Austin, TX United States

**Keywords:** correction, COVID-19, physicians, misinformation, infodemic, infodemiology, nurses, social media

## Abstract

**Background:**

Health misinformation is a public health concern. Various stakeholders have called on health care professionals, such as nurses and physicians, to be more proactive in correcting health misinformation on social media.

**Objective:**

This study aims to identify US physicians’ and nurses’ motivations for correcting health misinformation on social media, the barriers they face in doing so, and their recommendations for overcoming such barriers.

**Methods:**

In-depth interviews were conducted with 30 participants, which comprised 15 (50%) registered nurses and 15 (50%) physicians. Qualitative data were analyzed by using thematic analysis.

**Results:**

Participants were personally (eg, personal choice) and professionally (eg, to fulfill the responsibility of a health care professional) motivated to correct health misinformation on social media. However, they also faced intrapersonal (eg, a lack of positive outcomes and time), interpersonal (eg, harassment and bullying), and institutional (eg, a lack of institutional support and social media training) barriers to correcting health misinformation on social media. To overcome these barriers, participants recommended that health care professionals should receive misinformation and social media training, including building their social media presence.

**Conclusions:**

US physicians and nurses are willing to correct health misinformation on social media despite several barriers. Nonetheless, this study provides recommendations that can be used to overcome such barriers. Overall, the findings can be used by health authorities and organizations to guide policies and activities aimed at encouraging more health care professionals to be present on social media to counteract health misinformation.

## Introduction

### Background

Health misinformation is defined as any health-related claim of fact that is false based on the current scientific consensus [1]. It is a threat to public health because it impairs individuals’ ability to make appropriate health decisions, resulting in poor health behaviors and outcomes [2]. For instance, research has shown that exposure to misinformation, wherein tobacco and alcohol consumption protects people from COVID-19, is associated with greater tobacco and alcohol consumption [3]. Similarly, researchers found that beliefs about COVID-19 misinformation are associated with lower COVID-19 knowledge and lower adherence to preventive behaviors [4].

Various stakeholders have noted that social media is a fertile ground for health misinformation, and interventions are needed to correct it [5-7]. With the global spread of COVID-19, the United Nations [8] and World Health Organization [9] have emphasized that health misinformation, particularly on social media, is a public health threat that needs to be addressed. An intervention that the United Nations [8] has proposed is the formation of digital first responders—volunteers on social media, whose role is to share correct information and, to some extent, correct health misinformation.

Among social media users, health care professionals, particularly physicians and nurses, may serve as role models in correcting health misinformation on social media as they possess clinical knowledge they can share with the public. Research suggests that physicians and nurses have good levels of eHealth literacy [10-12], which enables them to select and share correct web-based health information with the public. Moreover, nurses and physicians are trustworthy sources of health information as they belong to the top US professionals considered honest and ethical [13]. Some health care professionals also have a strong social media following [14] that can be leveraged to amplify the communication of accurate health information on social media. This is evidenced by recent media [15-17] and scholarly [18,19] reports of physicians and nurses who are also social media influencers. Furthermore, research suggests that physicians and nurses tend to have a positive attitude toward using social media professionally as it can improve one’s knowledge [20] and facilitate health information sharing among colleagues [21] and the public [22,23].

Despite how well positioned health care professionals are for correcting health misinformation on social media, empirical studies on their motivations and barriers in performing such an act are missing. To date, relevant literature is limited on encouraging health care professionals to be on social media to help correct health misinformation [24-26]. For instance, O’Connor and Murphy [24] encouraged health care professionals to rebut misleading health information on social media by using appropriate sources. Rubin [25] noted that a crucial step in correcting health misinformation is for health care professionals to have a social media presence. Swire-Thompson and Lazer [26] also encouraged health communicators, particularly health care professionals, to correct health misinformation on social media as research suggests that such corrections can prevent people from believing misinformation.

### Objectives

If health authorities and organizations would like to encourage health care professionals to be on social media and become digital first responders, it is necessary to understand why health care professionals want to do it and identify barriers that they might face in correcting health misinformation on social media. As part of a larger study on the role of health care professionals in correcting health misinformation [23], this study aims to answer the following three research questions:

- Research question 1: what motivates health care professionals to correct health misinformation on social media?
- Research question 2: what barriers do health care professionals face when correcting health misinformation on social media?
- Research question 3: what are health care professionals’ recommendations to overcome barriers in correcting health misinformation on social media?

## Methods

### Participant Selection

Target participants included US physicians and registered nurses. We focused on physicians and registered nurses as they form the largest group of health care professionals in the United States [27] and are reported by the media as an emerging group of social media influencers [15-17]. In addition, a 2020 Gallup poll showed that these health care professionals are considered the most honest and ethical professionals in the United States [13]. Besides being a licensed physician or registered nurse in the United States, other eligibility criteria included working as a physician or registered nurse for at least a year and being an active social media user.

A combination of purposive (ie, active social media users with active US physician or registered nurse licensure and with ≥1 year of work experience) and snowball sampling strategies (ie, asking for referrals and social media hopping) were used to recruit participants. We communicated with potential participants by sending an email or direct message to their social media accounts on platforms such as Facebook, Twitter, Instagram, and LinkedIn. To achieve maximum variation sampling (ie, recruiting diverse participants to obtain multiple perspectives) [28], we recruited participants from various age groups, sex, and practice areas. This study was approved by the institutional review board of the University of Texas at Austin (2019-10-0149). Participants provided written and verbal consent before the data collection.

### Data Collection

Semistructured interviews were conducted between January and March 2020 via video conferencing platforms (ie, Zoom [Zoom Video Communications] or Skype [Microsoft Corporation]) or mobile phone calls. An interview guide was used during the semistructured interviews, which provided the ability to explore insights based on interviewees’ responses to questions within the interview guide. Considering that the results presented here are part of a larger qualitative study on health misinformation, the following interview questions were relevant to this study:

- As a health care professional, do you think that you have the responsibility to correct health misinformation on social media? Why?
- What do you think are barriers for health care professionals to correct health misinformation on social media?
- What suggestions or advice can you give to health care professionals when correcting health misinformation on social media?

The interviews were conducted by JRB (first author). The interviewer had the relevant qualifications to conduct the study as he had degrees in nursing (bachelor’s), public health (master’s), and communication science (doctorate). In addition, he also published health informatics–related articles based on interview data. Participants did not know the interviewer personally or professionally before recruitment. The interviews lasted an average of 21.69 (SD 6.43) minutes and were audio recorded. Participants were given a US $20 gift voucher as an incentive.

### Participants and Characteristics

We invited 212 health care professionals, of whom 30 (14.2% response rate) agreed to participate. The sample was composed of 50% (15/30) physicians and 50% (15/30) registered nurses. The sample size was sufficient for this study based on the advice of Green and Thorogood [29] that rich insights for qualitative work can be obtained after interviewing 20 participants.

Table 1 shows the characteristics of the participants. Most of the 30 participants were women (20/30, 67%). Their ages ranged from 27 to 65 years (mean 43.8 years, SD 9.73), and their work experience as a health care professional ranged from 6 to 40 years (mean 18.05 years, SD 9.69). They came from a variety of practice areas, including pediatrics (5/30, 17%), pediatric nursing (4/30, 13%), public health nursing (3/30, 10%), cardiology (2/30, 7%), emergency medicine (2/30, 7%), and oncology (2/30, 7%). Participants were located in 16 US states, with most practicing in Texas (7/30, 23%) and Pennsylvania (4/30, 13%). Although all participants were using multiple social media platforms (eg, Facebook, Twitter, LinkedIn, and Instagram), all were active Twitter users for the past 6.85 years on average (SD 2.85). All had experienced correcting health misinformation on social media.

**Table 1:** Participant characteristics (N=30).

Characteristics			Values
**Sex, n (%)**			
	Female	20 (67)	
	Male	10 (33)	
Age (years), mean (SD; range)			43.8 (9.73; 27-65)
Number of years using Twitter, mean (SD; range)			6.85 (2.85; 0.5-11)
**Health care profession, n (%)**			
	Registered nurse	15 (50)	
	Physician	15 (50)	
Number of years as a health care professional, mean (SD; range)			18.05 (9.69; 6-40)
**Practice areas^a,b^, n (%)**			
	Pediatrics	5 (17)	
	Pediatric nursing	4 (13)	
	Public health nursing	3 (10)	
	Cardiology/emergency medicine/oncology	2 (7)	
	Anesthesiology/cardiology nursing/critical care nursing/diabetes nurse consultant/epidemiology/family nurse practitioner/family medicine/float nursing/gastroenterology/hematology/internal medicine/psychiatry/rehabilitation medicine/resuscitation and innovation/school nursing/women’s health nursing	1 (3)	
**Practice location^b^, n (%)**			
	Texas	7 (23)	
	Pennsylvania	4 (13)	
	California/Maryland/New Jersey/Utah/Wisconsin	2 (7)	
	Colorado/Georgia/Illinois/Louisiana/Missouri/New Mexico/New York/North Carolina/Ohio	1 (3)	

^a^Some participants had multiple specializations.

^b^Count per item.

### Data Analysis

We transcribed the audio recordings and interview notes after each interview. The resulting transcripts and interview notes were uploaded to MAXQDA 2018 (VERBI GmbH) for qualitative data analysis. The data analysis was guided by a phenomenological perspective to thematic analysis [30], considering that the interview data contained participants’ perspectives and experiences about their motivations, barriers, and recommendations to correct health misinformation on social media.

Initially, we performed an iterative process of open (ie, to break down data into smaller analytical points) and axial coding (ie, grouping open codes to generate connections between categories and subcategories) to uncover themes and subthemes [28]. Codes were derived from the data (ie, a priori) and classified under themes (ie, motivations, barriers, and recommendations) and subthemes (eg, personal motivations, intrapersonal barriers, and build a social media presence). Table 2 provides the coding tree.

**Table 2:** Coding tree (N=30).

Themes and subthemes				Codes (participants per profession), n (%)		
				Physician (n=15)		Registered nurse (n=15)
**Motivations to correct health misinformation on social media**						
	**Personal motivations**					
		Personal choice	1 (7)		1 (7)	
		Urge to correct people	3 (20)		0 (0)	
	**Professional motivations**					
		Stand up for what is right as a health care professional	6 (40)		6 (40)	
		Keep people safe	1 (7)		5 (33)	
		Opportunity to educate more people	4 (27)		1 (7)	
**Barriers in correcting health misinformation on social media**						
	**Intrapersonal barriers**					
		Lack of positive outcome	10 (67)		7 (47)	
		Lack of time	9 (60)		3 (20)	
		Lack of self-efficacy	3 (20)		3 (20)	
		Avoidant behavior	1 (7)		4 (27)	
		Lack of voice to influence others	2 (13)		2 (13)	
		Difficulty in producing social media content	3 (20)		0 (0)	
	**Interpersonal barriers**					
		Harassment and bullying	14 (93)		9 (60)	
		Difficulty to have a meaningful conversation on the web	13 (87)		6 (40)	
	**Institutional barriers**					
		Lack of organizational support	6 (40)		4 (27)	
		Lack of social media training	3 (20)		1 (7)	
**Recommendations to overcome barriers in correcting health misinformation on social media**						
	**Get misinformation and social media training**					
		Be familiar with the literature and collate resources	2 (13)		6 (40)	
		Learn to use social media professionally	2 (13)		2 (13)	
		Connect with role models or mentors	2 (13)		0 (0)	
		Learn how to correct misinformation	1 (7)		0 (0)	
	**Build a social media presence**					
		Be on social media	11 (73)		1 (7)	
		Disseminate facts	6 (40)		5 (33)	
		Build an audience	5 (33)		1 (7)	
		Be part of a community	3 (20)		1 (7)	
		Maintain professionalism	2 (13)		0 (0)	

A total of 3 coders (1 registered nurse, 1 medical student, and 1 information studies graduate student) independently coded a sample of the transcripts. The results showed good interrater reliability (Krippendorff α=.82). After preliminary coding, the research team discussed the codes and resulting themes and subthemes to check whether data saturation was achieved. After several meetings, the research team deemed that data saturation was achieved based on the presence of well-developed and interrelated themes and subthemes. In addition, all codes were accounted for in a particular theme or subtheme, and no new codes could be derived from the data [28].

### Trustworthiness

This study adheres to qualitative trustworthiness by observing the principles of credibility, transferability, dependability, and confirmability [31]. We promoted credibility by establishing rapport with the participants to obtain honest remarks and by using iterative questioning to clarify the details. We enhanced transferability by upholding maximum variation sampling (eg, interviewing health care professionals of different ages, sexes, and practice areas). The study was also dependable as the research team followed the approved research protocol. Finally, the results were confirmed by providing anonymous quotes from participants to support our findings.

### Reflexivity Statement

The study team was composed of researchers with expertise in health information interaction, information quality, and qualitative research. JRB has 8 years of research experience in these areas, whereas YZ and JG have >10 years of experience. All study team members are social media users and are aware of the negative implications of health misinformation on public health. The research team ventured on health misinformation research in 2019 because of a fellowship awarded to JRB; thus, the research would have been conducted regardless of the COVID-19 pandemic. Nonetheless, this study became much more relevant because of the infodemic that was brought about by the pandemic. JRB was motivated to conduct this study because, as a nurse in the Philippines, he believes that health care professionals can leverage social media to correct health misinformation. Although JRB’s belief can present a bias in this study, he maintains neutrality by avoiding agreement or disagreement (verbally and nonverbally) with the participants’ statements during interviews and by being self-aware of his biases during data analysis. To further minimize bias, we recruited participants who were not part of our personal or professional network.

## Results

### Motivations to Correct Health Misinformation on Social Media

Participants identified several personal and professional motivations to correct health misinformation on social media.

#### Personal Motivations

There were two personal motivations associated with correcting health misinformation on social media. Some (physician: 1/5, 7%; registered nurse: 1/15, 7%) noted that it is a personal choice because they think that it is not their legal obligation to correct health misinformation:

Legally speaking, I don’t have any obligation to correct or actively correct health misinformation online or even participate on social media. But I chose to do so because I feel that it’s probably about it. And know there are no incentives for anyone to necessarily jump to social media to do this but some of us try to do this.

physician 1

Similarly, some (physician: 3/15, 20%) noted that they might have the urge to correct people because that seems to be a reflex for them as health care professionals:

I will say though, I don’t know if it’s a duty, I think it’s almost a reflex for a clinician to encountering misinformation online to respond and correct it if they have the time and inclination.

physician 11

#### Professional Motivations

There were three professional motivations associated with correcting health misinformation on social media. For instance, some (physician: 6/15, 40%; registered nurse: 6/15, 40%) noted that as health care professionals, they need to correct health misinformation on social media because it is an act of standing up for what is right:

Because people look to us as experts in these areas and if we are not standing up and making clear what is accurate information and what’s not, I personally feel that we’re not doing our job. If we are not debunking misinformation, then it’s detrimental to the health of all our community members.

registered nurse 13

Others (physician: 1/15, 7%; registered nurse: 5/15, 33%) considered correcting health misinformation on social media as part of their professional responsibility to keep people safe against the ill effects of health misinformation:

I think that when we entered this profession and took an oath to do no harm...and if we are allowing health misinformation to run wild out there, especially for our own patients, allowing that information to continue to have an effect is going against what were here to do or were here to achieve.

physician 1

A few (physician: 4/15, 27%; registered nurse: 1/15, 7%) noted that such an act is an opportunity to educate more people as social media opens interactions to a global community of health information seekers:

50-60 years ago when physicians were trained, they were taught to educate their neighbors, their patients and their community as well as treating people. So now, our community has become a global community. So, I believe that we, as physicians, have the responsibility to educate using whatever medium to reach the largest number of people. Because people are so interconnected and the way that individuals obtain information and get misinformation has changed quite a bit a few years ago.

physician 13

### Barriers in Correcting Health Misinformation on Social Media

Participants pointed out several barriers that they face to correct health misinformation on social media. Broadly, these barriers can be categorized as intrapersonal, interpersonal, and institutional.

#### Intrapersonal Barriers

Many (physician: 10/15, 67%; registered nurse: 7/15, 47%) noted that health care professionals might be discouraged from correcting health misinformation on social media because they do not see the immediate positive change that results from it:

I think another barrier online is that it just feels like you are fighting an endless battle because you don’t ever see the progress that’s being made. I’m lucky enough to have a big enough platform that I actually get to see some of the benefit of it now and so it’s really gotten easier for me to do that because I see the difference that it’s making. But when you’re first starting out, it can just feel overwhelming like you don’t make any progress.

physician 4

Another intrapersonal barrier was the lack of time. Several (physician: 9/15, 60%; registered nurse: 3/15, 20%) participants noted that given their current clinical workload and other responsibilities, some health care professionals may not have the time to correct health misinformation on social media:

Some physicians have the barrier that they just don’t have the time. We already have so many demands on our time and physicians just don’t have the time to do it and don’t want to spend whatever precious time they have going through this.

physician 13

Some (physician: 3/15, 20%; registered nurse: 3/15, 20%) also noted a lack of self-efficacy in correcting health misinformation on social media. For instance, health care professionals may not have the specialist knowledge to detect health misinformation and the training to effectively correct it:

They just might not know that the information is incorrect themselves or they might not know enough about the truth or the facts to be able to dissuade someone whose sharing falsehoods or falsities. I think that’s a major one.

registered nurse 11

Others (physician: 1/15, 7%; registered nurse: 4/15, 27%) also noted that some health care professionals may have an avoidant behavior where they would prefer to avoid any confrontation and arguments arising from correcting others or they may not feel comfortable being on social media at all:

They might not think of themselves as experts in whatever topic to be able to correct someone. They may not feel comfortable on social media or in person correcting people.

registered nurse 13

In addition, some (physician: 2/15, 13%; registered nurse: 2/15, 13%) lamented that their voices on social media might not necessarily be heard because they lack the influence to enact changes (eg, few social media followers):

Unfortunately, the loudest voices are heard. It would be great if the nurses, the largest population of health professionals, our voice could have been louder. I feel just because of our sheer number and it could have drowned out the bad health information it could have. But that’s not the reality that we’re in.

registered nurse 7

Finally, a few (physician; 3/15, 20%) noted that producing content (eg, conducting research for the correction, crafting the message, and adding images or videos) to correct health misinformation on social media takes time and considerable expertise that serves as a barrier:

Authoring and making content take a lot of time. It takes some skill, it takes writing capacities, it takes communication skills, it takes preparation, if it’s video it takes lighting and makeup. No matter how silly that sounds but there’s a lot of work involved in that at times.

physician 10

#### Interpersonal Barriers

Many (physician: 14/15, 93%; registered nurse: 9/15, 60%) pointed out that health care professionals are at risk of being bullied and harassed by other social media users as they correct health misinformation. Given that correcting others may result in heated debates and arguments, some participants have experienced bullying and harassment, such as being accused as child predators, conspiring with pharmaceutical companies, and receiving negative reviews and mob attacks on the web:

Every single time you post about vaccines you will get harassed if your platform is large enough that people will see it. I’ve had times where I just post CDC statistics on how many people die from influenza each year and end up having to make all of my accounts private because I get such a vast influx of people just attacking. I’ve had people come on to my Instagram and comment on pictures of my children saying that they look vaccine-injured and that I am a child abuser and that I’m in bed with big pharma and my kids should be taken away and CPS [Child Protective Services] should be called.

physician 4

Bullying and harassment are carried out by social media users with whom the participants are not familiar, such as trolls who operate under the veil of anonymity. For some participants, such negative experiences may deter health care professionals from correcting health misinformation on social media. Although some participants ignored trolls as a means of coping with bullying and harassment, some had to make their social media accounts private, limit interactions, block people, or stop engaging on social media:

It was not pleasant [experiencing bullying and harassment] and what I basically did was I just disengaged, and I didn’t go back to the post. It did not make me feel good. A matter of fact, it made me feel really disgusted with that elected official, that he would behave that way.

registered nurse 10

Another interpersonal barrier was the difficulty of having a meaningful conversation on the web. Most (physician: 13/15, 87%; registered nurse: 6/15, 40%) preferred face-to-face interactions when correcting health misinformation as interactions on the internet remove vital verbal and nonverbal cues that are needed to establish rapport and the relationship required to dispel misinformation. Moreover, as social media users can opt for anonymity, the conversations may not be as fruitful and respectful compared with face-to-face interactions, such as during patient visits, where effective health education sessions can occur:

The problem I have with online discourse is this: virtually no tone. It’s very difficult unless you’re using all caps and exclamation marks to communicate tone on Twitter for instance. Twitter being so short you can come across as curt even if you did not intent to be. Whereas face-to-face, you get all the nonverbal cues, facial expression, sometimes even touch when appropriate.

physician 11

#### Institutional Barriers

Institutional barriers were also identified by the participants. For instance, several participants (physician: 6/15, 40%; registered nurse: 4/15, 27%) noted a lack of organizational support for correcting health misinformation on social media. This stems from the lack of institutional backing for health care professionals to be on social media because of privacy concerns:

So primarily, a lot of physicians don’t feel comfortable [being on social media]. For years, the health care system has told physicians not to go on social media because of patient privacy and the variety of other issues.

physician 13

To distance themselves from their employers, a few participants tended to write a statement in their social media profiles, particularly on Twitter, that their opinion was their own and not representative of their employer or institution:

They don’t engage [in correcting health misinformation on social media] because, I think, maybe some [health care] professionals are afraid to do it because of the organization they work in. I don’t list my organization on Twitter because I don’t have enough characters to do it, and also I put a disclaimer that the opinions or mine and a retweet doesn’t mean I endorse something. So, I have some disclaimers.

registered nurse 10

Although a participant noted that, through the years, “a lot of [health care] organizations are really asking their clinicians to be on social media*”* [physician 15], there is still a lot of work for health care institutions to support their health care professionals as they create a social media presence. In addition, institutions tend not to provide incentives for health care professionals who correct health misinformation on social media:

Like I said, there are no [institutional] incentives for anyone to participate. It’s really self-driven.

physician 1

Another institutional barrier was the lack of social media training. Some (physician: 3/15, 20%; registered nurse: 1/15, 7%) noted that they just learned to use social media professionally during their practice:

We don’t get a lot of training on this [social media training] so everybody just makes it up as we go. I think there’s more and more an effort to get physicians exposed to best practices and to literature about what’s an effective way to communicate. With that, it’s still early and it doesn’t always penetrate into the entire workforce.

physician 15

In addition, formal training in using social media professionally was usually not part of the health care professionals’ curriculum and clinical training:

None of us get this training in our training programs, on how to use media and social media. So, in correcting people online, I think, first off, there’s oftentimes no formal training. People do this just because they often enter into social media, just using it on their own. And then there’s just general communication training to which I think we don’t really receive a lot of it in both nursing and physician training programs.

physician 6

### Recommendations to Overcome Barriers in Correcting Health Misinformation on Social Media

To overcome some of the barriers in correcting health misinformation on social media, participants recommended that health care professionals get misinformation and social media training and build their social media presence.

#### Get Misinformation and Social Media Training

For correcting health misinformation, some participants (physician: 2/15, 13%; registered nurse: 6/15, 40%) noted that it is crucial to be familiar with the literature (eg, up-to-date literature about a specific health issue or condition) and collate resources that can be disseminated when correcting health misinformation. Health care professionals should always project an image of expertise, which can be accomplished by having a command of the literature and resources that are specific to a health topic or issue:

I think that we should be careful in our response to show that we’re knowledgeable. Don’t respond to something if you don’t know what you’re talking about. That’s just going to make the situation worse. But when it’s your content area for instance and you know the information is wrong, address it right away. Make sure that you are knowledgeable about what you’re saying. But then also provide the person in question with resources to show them that you’re not just making something up, you’re not like we say talking out of the side of your neck but you actually have evidence to support what it is that you’re saying.

registered nurse 11

Some (physician: 2/15, 13%; registered nurse: 2/15, 13%) also recommended that health care professionals learn how to use social media professionally. Although institutional training may be limited or unavailable, there are several professional groups (eg, Association for Healthcare Social Media and Doctors on Social Media) that health care professionals can join in to start learning about professional social media use (eg, what to post, creating engaging graphics and videos, and responding to health misinformation):

If health care professionals want to do it [correcting health misinformation on social media], they shouldn’t go into it without any kind of [professional social media] training or support. They are very likely to run into harm, they can have their reputation harmed, they can have their job threatened, there’s a lot of risks to doing it.

physician 9

Few (physician; 2/15, 13%) also noted that it is important to connect with role models or mentors who can advise when correcting health misinformation on social media. Typically, an ideal mentor has a strong social media presence (eg, high social media followers) and is an opinion leader (eg, their posts are shared by many followers):

I would tell them to look at the people that have already done it successfully. If they want to speak out on a health issue, see the main experts that are speaking out and kind of see how they are doing it and then be comfortable and then start speaking out for themselves.

physician 7

A participant (physician; 1/15, 7%) also noted that it is crucial for health care professionals to understand what misinformation is and the means to correct it:

Physicians operate under the assumption that there is an information deficit, this is incorrect. Generally, we’re used to people coming to acquire information and being open and receptive to information. The problem is that disinformation is not an information deficit, the problem is that disinformation represents a glut of misinformation. So, you can’t simply counter it by providing the correct information. Everybody has the correct information available to them. It’s on Google and it’s not far away. What physicians need to do and what they will always fail to do to correct misinformation and disinformation until they recognize it is that it’s not a matter of just telling people what the reality is, you have to reach them from a point of personal identity, a personal relationship. You have to create cause for spread of disinformation and you basically have to treat it like an information war like a propaganda war and not ‘I hope these people just lack information or are ignorant.’ They are not ignorant.

physician 9

#### Build a Social Media Presence

After obtaining relevant training, participants also recommended that health care professionals build their social media presence. The first step is to be on social media. For instance, many (physician: 11/15, 73%; registered nurse: 1/15, 7%) participants noted that it is crucial for health care professionals to have a professional social media account like Twitter because it is a good platform for publicly receiving, sharing, and discussing relevant health information:

It’s really important that people [health care professionals] engage in that they try to get on social media [like Twitter] to help educate the entire world on important topics. We want to make sure that everybody is working together to keep the health of all of our people safe and keep everyone healthy. And we can do that by combatting all this misinformation that’s out there.

physician 13

After creating a social media account, several (physician: 6/15, 40%; registered nurse: 5/15, 33%) participants noted that health care professionals need to disseminate facts, which serves as a foundation for building an audience:

I think it probably starts with sharing good information. I don’t know if we can police everybody and correct all the bad information, but I think we really need to stand up as health care professionals and make sure that we are sharing good information so that people can come to us and know what’s right basically.

physician 3

In addition to using social media as a platform to share facts that might correct health misinformation, some (physician: 5/15, 33%; registered nurse: 1/15, 7%) participants noted that sharing content might also attract followers that can assist in building an audience. This is based on the belief that the more social media followers a person has, the more influential the person’s voice becomes when they enact change (eg, dispelling health misinformation):

It’s really difficult to disrupt things. You need to learn to use it [social media] effectively and build an audience because just being on there alone isn’t enough. You kind of have to know how to use it in a way that’s going to allow your audience to grow. Otherwise, you’re gonna just be talking to [few people] rather than talking to 200, 2000 or 2 million people.

physician 1

In addition, part of building an audience is to be part of a community of health care professionals on social media. A few (physician: 3/15, 20%; registered nurse: 1/15, 7%) noted that health care professionals can do this by using relevant hashtags (eg, #NurseTwitter and #MedTwitter) in their Twitter posts. By using hashtags as a means of social learning, health care professionals can become learners and mentors on how to correct health misinformation on social media (eg, having exposed to posts with a #NurseTwitter or #MedTwitter hashtag can provide examples on how to correct):

So, there’s a hashtag #NurseTwitter or #NursesRetweet or #NurseAcademics or whatever. I believe it sets an example for other nurse colleagues who may be new to Twitter or may not know how to respond to misinformation and then they could see by example that basically we just need to share the correct information but not engage in some big argument and get into some kind of dramatic engagement on Twitter and social media because it doesn’t do any good.

registered nurse 10

Finally, 2 participants (physician; 2/15, 13%) emphasized that health care professionals should maintain professionalism. This is evidenced by being respectful to others (regardless of how disrespectful others are) and providing credible evidence to any statement posted on social media:

Don’t get into fight with people. Maintain your professionalism. Make sure that whatever you’re saying, you’re saying it with evidence. That’s the most important thing. You don’t wanna get into kind of a back and forth tug of war with somebody who is just trying to goad you along. So, you just need to make sure that you are behaving in a professional manner and remember that you’re still being representative of your profession while you’re on social media. So, whatever you post just think ‘what would something I would say to that person’s face?’ If it is – then you are free to post it. If not, then don’t post it.

physician 13

## Discussion

### Principal Findings

**Figure 1:**
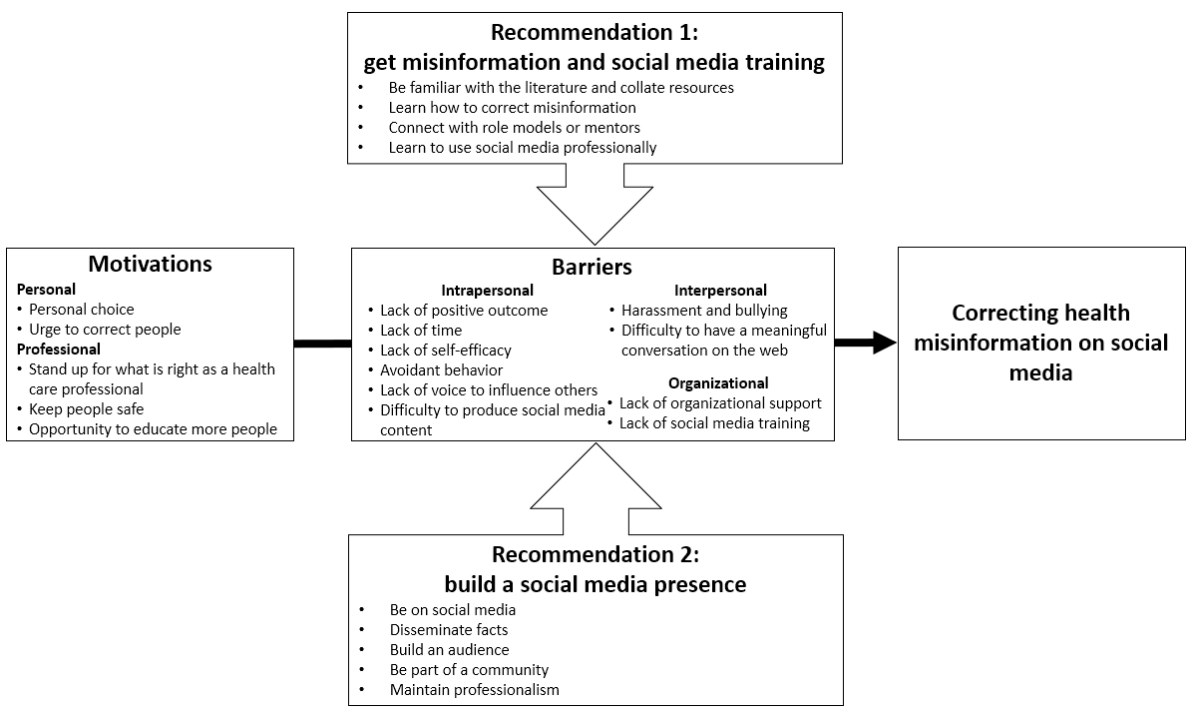
A model depicting US physicians’ and nurses’ motivations, barriers, and recommendations for correcting health misinformation on social media.

This qualitative study among 30 US physicians and nurses revealed several motivations, barriers, and recommendations related to correcting health misinformation on social media. Figure 1 shows a model that summarizes these findings.

In terms of motivations, we found that participants were motivated to correct health misinformation for both personal (ie, urge to correct people and personal choice) and professional (ie, stand up for what is right as a health care professional, opportunity to educate more people, and keep people safe) reasons. Although there is no legal mandate for them to correct health misinformation on social media, they are likely to be motivated by professional reasons. This is expected considering that correcting health misinformation on social media is an action that is compatible with their professional identity as health care professionals [32]. Specifically, correcting health misinformation is an opportunity for participants to demonstrate their clinical knowledge and skills with the intention of promoting health and doing good [32]. Besides, given their good levels of eHealth literacy [10,11] and positive attitudes toward social media [20-23], they are likely to leverage social media to demonstrate their professional identity.

This study also identified barriers for health care professionals to correct health misinformation on social media. A key contribution of this study is the grouping of barriers as intrapersonal, interpersonal, and institutional barriers. Intrapersonal barriers included lack of positive outcomes, lack of voice to influence others, lack of time, difficulty producing social media content, lack of self-efficacy, and avoidant behavior. Interpersonal barriers included harassment and bullying, as well as the difficulty of having a meaningful conversation on the web. Institutional barriers included lack of organizational support and lack of social media training. In general, the barriers identified are reminiscent of journalists’ barriers when correcting misinformation or disinformation [33,34]. Nonetheless, it is interesting to note that a serious consequence for both health care professionals and journalists who correct misinformation or disinformation on social media is harassment. Scholars have suggested that social media are breeding grounds for trolls and troublemakers [35], who can perpetuate several types of web-based harassment, such as cyberbullying, cyber-mob attacks, trolling, hateful speech, and web-based threats [36]. In fact, recent reports show that 1 in 4 US physicians experience personal attacks and sexual harassment on social media [37]. Research suggests that nurses experience cyberbullying and harassment, which can have a negative impact on their practice [38]. Thus, we argue that harassment is one of the greatest barriers to encouraging more physicians and nurses to correct health misinformation on social media, considering that they do not deserve such treatment when providing a voluntary service. As such, this finding serves as a call for health authorities and organizations to provide support (eg, institutional backing and providing social media training) when health care professionals decide to engage in misinformation correction activities.

In addition to motivations and barriers, participants also shared their recommendations on how health care professionals can overcome some of the barriers associated with correcting health misinformation on social media. First, they encouraged health care professionals to obtain misinformation and social media training by learning how to correct misinformation, being familiar with the literature and collating sources, learning to use social media professionally, and connecting with role models or mentors. In general, such recommendations point to the need to incorporate social media training as part of health profession education. Traditionally, communication training in health professions focuses on interpersonal communication between providers and patients and among providers [39]. With the global adoption of social media, there is a need to equip health care professionals with skills for effectively communicating health information in this channel [23-26,40]. Therefore, to effectively communicate with the public when correcting health misinformation on social media, in addition to interpersonal communication training, it is crucial to incorporate mass communication training, as social media is a hybrid of interpersonal and mass communication [41]. As such, this study calls for the reevaluation of communication training programs for health care professionals to effectively use social media for professional health communication. Such training is needed if we expect them to be on social media as health care professionals who can help correct health misinformation.

In addition to getting misinformation and social media training, participants recommended their peers build a social media presence by being on social media, disseminating facts, building an audience, being part of a community, and maintaining professionalism. Establishing a professional social media presence is needed to increase the probability of shaping the audience’s attitudes toward a specific issue [42]. In this study, participants highlighted the need to build a social media presence so that the corrections they post can be shared by many, which can then increase the chances that the correction can dispel misperceptions. To date, several organizations are helping health care professionals establish a social media presence. For instance, health care social media organizations, such as Doctors on Social Media [43] and the Association for Healthcare Social Media [44], provide support and training for nurses and physicians to improve their social media presence and effectively correct health misinformation. Furthermore, YouTube announced that it would provide support to health care professionals to increase their social media presence as a strategy to combat health misinformation [45].

### Limitations

This study has two limitations. First, participants in this study were represented by physicians and registered nurses. Although they comprise most of the US health care workforce [27], insights from other health care professionals (eg, dentists, pharmacists, and physical therapists) can be added in future studies. Second, the findings were derived from interviews with US participants. Hence, the findings may not be fully comparable with the experiences of health care professionals based outside the US. Future cross-country studies are needed to determine whether other factors (eg, perceived practice autonomy and perceived authority) could play a role in motivating health care professionals to correct health misinformation on social media.

### Conclusions

Given how widespread health misinformation is on social media (as demonstrated by the COVID-19 infodemic), health care professionals can lend their time to mitigate this public health concern. In this study, we found that US physicians and nurses are professionally and personally motivated to correct health misinformation on social media despite some of the barriers they face in performing such an act. It also sheds light on specific recommendations to minimize or overcome such barriers. In general, the findings can be used by health authorities and educational institutions when developing campaigns or educational programs to train health care professionals to correct health misinformation on and off social media.
